# SPICA: Swiss portal for immune cell analysis

**DOI:** 10.1093/nar/gkab1055

**Published:** 2021-11-08

**Authors:** Massimo Andreatta, Fabrice P A David, Christian Iseli, Nicolas Guex, Santiago J Carmona

**Affiliations:** Ludwig Institute for Cancer Research, Lausanne Branch, and Department of Oncology, CHUV and University of Lausanne, Epalinges 1066, Switzerland; Swiss Institute of Bioinformatics, Lausanne, Switzerland; Bioinformatics Competence Center, École Polytechnique Fédérale de Lausanne, CH-1015 Lausanne, Switzerland; Bioinformatics Competence Center, École Polytechnique Fédérale de Lausanne, CH-1015 Lausanne, Switzerland; Bioinformatics Competence Center, University of Lausanne, CH-1015 Lausanne, Switzerland; Ludwig Institute for Cancer Research, Lausanne Branch, and Department of Oncology, CHUV and University of Lausanne, Epalinges 1066, Switzerland; Swiss Institute of Bioinformatics, Lausanne, Switzerland

## Abstract

Single-cell transcriptomics allows the study of immune cell heterogeneity at an unprecedented level of resolution. The Swiss portal for immune cell analysis (SPICA) is a web resource dedicated to the exploration and analysis of single-cell RNA-seq data of immune cells. In contrast to other single-cell databases, SPICA hosts curated, cell type-specific reference atlases that describe immune cell states at high resolution, and published single-cell datasets analysed in the context of these atlases. Additionally, users can privately analyse their own data in the context of existing atlases and contribute to the SPICA database. SPICA is available at https://spica.unil.ch.

## INTRODUCTION

Recent breakthroughs in single-cell technologies have enabled the exploration of immune responses across diseases, tissues and individuals. Importantly, we have now the opportunity to discriminate specific immune states at single cell resolution, and to associate these states to key clinical parameters, such as responsiveness to immunotherapy and disease prognosis (1,2). However, interpretation of single-cell transcriptomics data in the context of previous studies and prior knowledge is challenging because of inconsistent cell state definitions. To address this problem, we have recently developed a novel analytical approach to interpret immune cell states from single-cell RNA sequencing (scRNA-seq) data (3). This framework uses cell type-specific ‘reference atlases’ to represent current knowledge for a specific cell type at high resolution. New datasets, not included in the atlases, can then be analysed and annotated by projection onto one of the atlases.

The Swiss portal for immune cell analysis (SPICA) aims to provide researchers a reference web resource to explore immune cell states in mouse and human. SPICA provides easy exploration of cell type-specific reference atlases, and a database of published studies analysed in the context of these atlases. Users can also privately upload their single-cell datasets and project them onto one of the atlases, to obtain an automated analysis of immune cell state composition and gene expression profiles across samples or conditions.

## RESULTS

**Figure 1.**
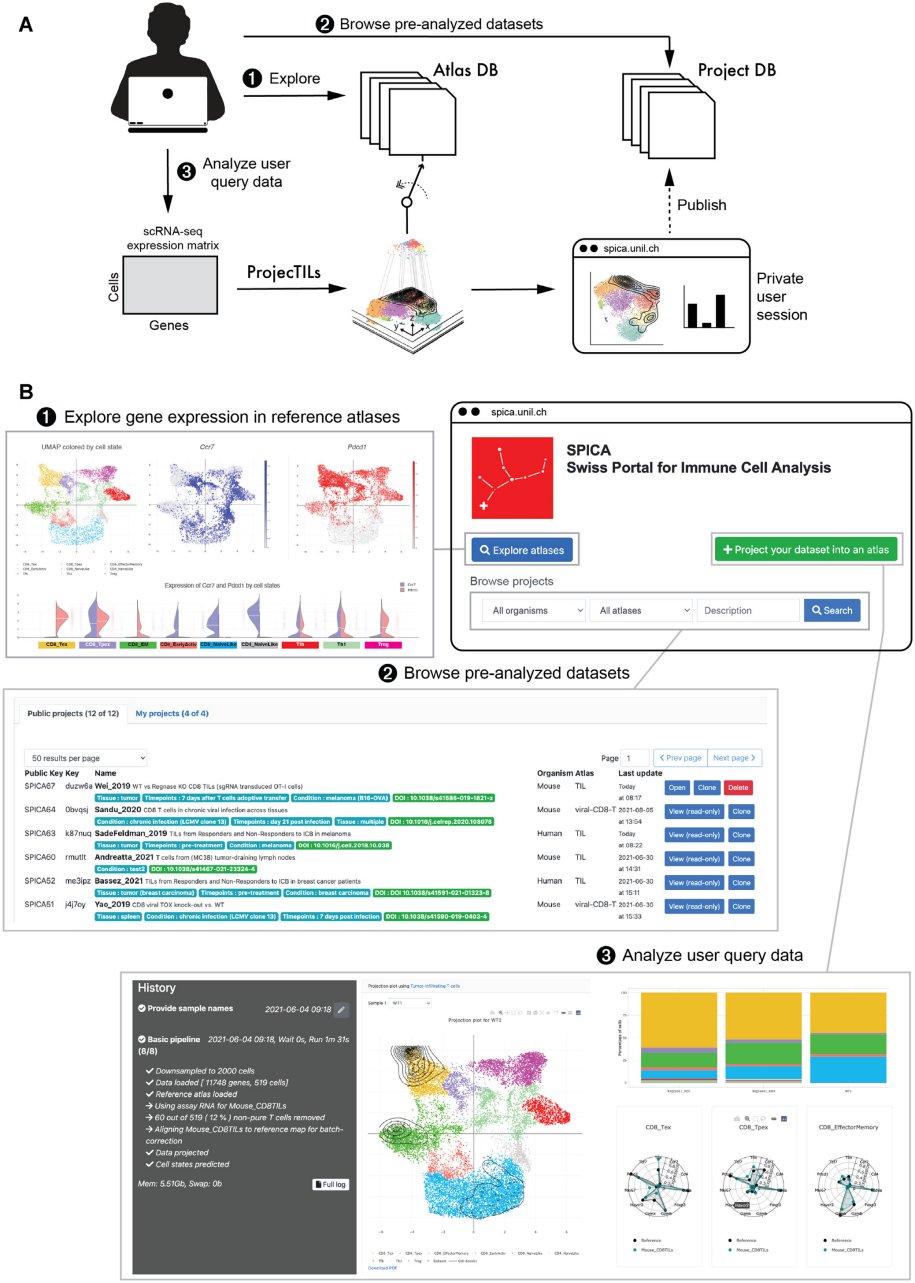
Overview of SPICA. (**A**) Three main entry points are accessible to the user: (1) a database of curated reference immune cell atlases; (2) a database of pre-analysed datasets (‘projects’), derived from published single-cell transcriptomics studies and (3) an interface for the analysis of new single-cell data uploaded by the user. Analyses of user-uploaded data are kept confidential in a private session, unless explicitly made public by the user. (**B**) The three main components of SPICA are accessible from the portal webpage, and allow (1) exploring gene expression from reference atlases, (2) browsing the database of public (or private), pre-analysed datasets and (3) projecting user query data to analyse them in the context of one of the available reference atlases.

The SPICA portal consists of three main components (Figure 1A): (i) a database of interactive, curated immune cell reference atlases; (ii) a searchable database of pre-analysed datasets, allowing the comparison of immune cell states in different studies and conditions; (iii) an interface for the projection of new data, enabling the analysis of user query datasets in the context of existing reference atlases. In the following section, we briefly describe these three main SPICA components. Next, we illustrate some case studies.

### Exploring reference atlases

Accessing the ‘Explore Atlas’ functionality in SPICA, the user can interrogate one of the existing reference atlases in the database (Figure 1B). On the page for pairwise gene comparison, two UMAP plots allow visualizing the expression pattern of two user-selected genes in the same reference space. To facilitate quantitative comparison of gene expression for the two selected genes in different cell subtypes, a series of split violin plots are also provided. The ‘Multiple gene comparison’ page enables the joint visualization of expression patterns for multiple genes, both for individual cells on the UMAP space, as well as in terms of average expression for the cell types contained in the atlas.

### Browsing pre-analysed datasets

SPICA hosts a collection of public scRNA-seq datasets of mouse and human immune cells from different tissues and diseases, pre-projected onto a relevant reference atlas (see next section). A search bar allows filtering the database selection by organism, by atlas type, or by a free text description (Figure 1B). Each pre-analysed dataset (a ‘project’) can be investigated online or downloaded for downstream analysis. For advanced users, an API is available to interact with SPICA programmatically (https://spica.unil.ch/home/api).

### Projecting user query data onto reference atlases

Besides pre-analysed datasets, users can apply SPICA to interpret their own scRNA-seq data by reference atlas projection. Raw or normalized gene expression matrices (typically UMI counts or TPM, transcripts per million) can be uploaded in several formats (tabular gene-cell matrices, 10× MEX format, hdf5 format, Seurat .rds R objects), with the possibility to upload multiple samples at once as a compressed archive. After selecting a reference atlas, the user query data will be projected onto the atlas using ProjecTILs (3). This algorithm corrects for batch effects and embeds query data in the low-dimensional spaces of the atlas, allowing users to visualize their data and to compare them in the same system of coordinates as the annotated reference atlas. For each sample included in a project, the projected query cells are overlaid on the UMAP representation of the reference atlas, and their density shown as contour lines (Figure 1B). Cell subtype annotations of the query data are assigned by ProjecTILs, and summarized as stacked barplots, facilitating the comparison of cell subtype composition of different samples. Radar plots for a customizable panel of genes allow qualitatively assessing whether the expression profiles of query samples differ from each other or from the reference (Figure 1B and interactive at https://spica.unil.ch/projects/Carmona_2019). Within the analysis session, the user can also perform differential expression (DE) analysis between a pair of samples, for a specific cell subtype. DE analysis results are reported in an exportable tabular form, indicating log-change and p-values for differentially expressed genes, and displayed as interactive volcano plots. All plots can be exported in high-resolution PDF format. Full project sessions including plots and input and output data can also be downloaded. Note that user-uploaded datasets are kept confidential within a private session, and require explicit ‘publishing’ permission from the user to become publicly available on the portal.

In the following section we present two case studies using publicly available scRNA-seq datasets to highlight the functionalities of the database.

## CASE STUDIES

### Associating T cell phenotypes to immunotherapy responsiveness

**Figure 2.**
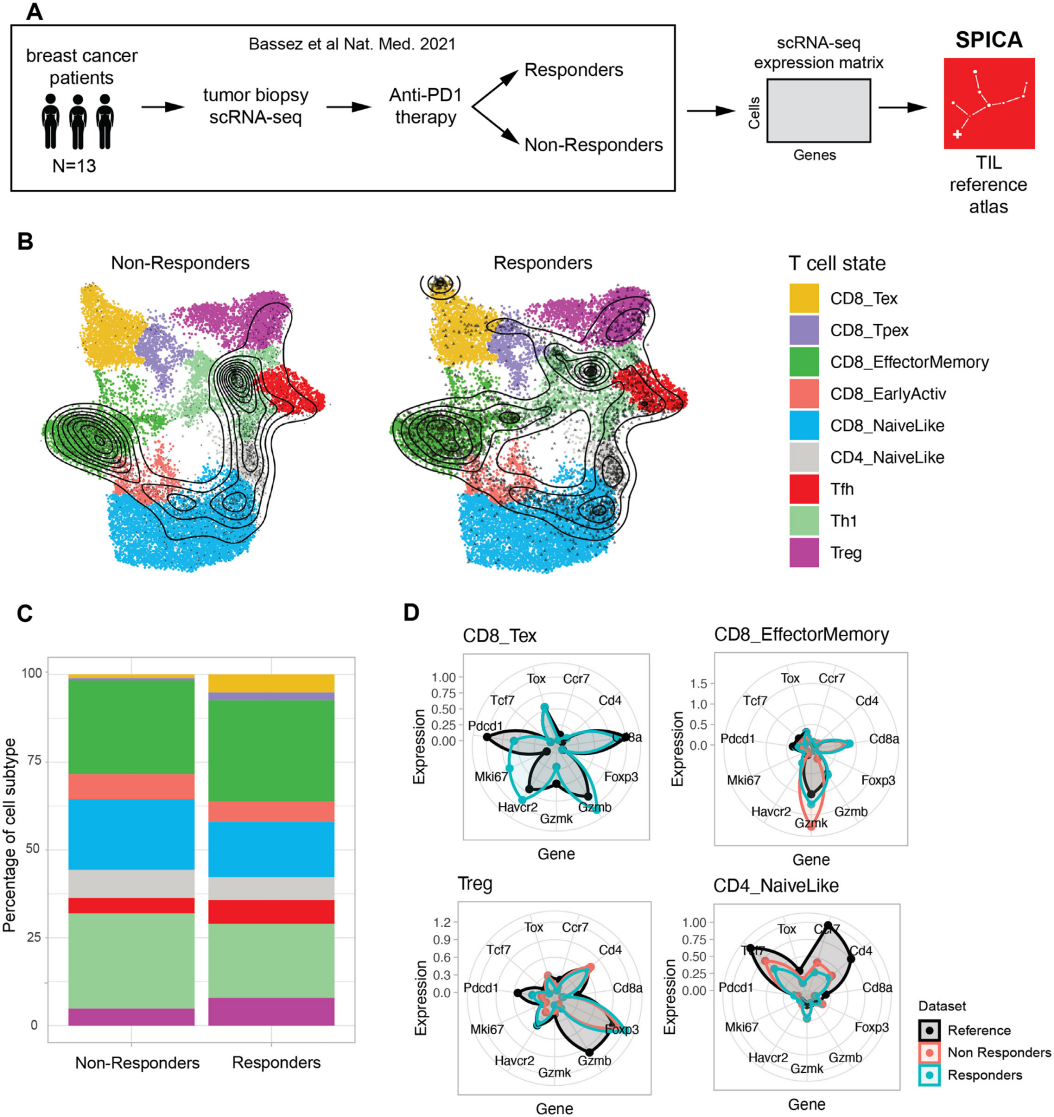
Case study of tumor biopsies. (**A**) Overview of the tumor scRNA-seq data generated by Bassez et al. of patients that responded or did not respond to immunotherapy. For SPICA analysis, only samples with at least 500 T cells were considered (*N* = 13, of which 7 correspond to responders and 6 non-responders). (**B**) UMAP plots of the reference TIL atlas; black points represent projected query cells, contour lines represent the density of projected cells. (**C**) Cell subtype composition (percentage) for each group of samples. (**D**) Gene expression profiles of projected cells and reference profiles for different cell subtypes. For each subtype, only samples with a pre-defined minimum number of cells are plotted. This case study can be explored online at https://spica.unil.ch/projects/Bassez_2021.

In a recent study, Bassez *et al.* (4) performed scRNA-seq on tumor biopsies from breast cancer patients, taken immediately before (pre-treatment) and after (on-treatment) receiving anti-PD1 immunotherapy. To investigate whether there is an association between pre-treatment T cell subtype composition and responsiveness to therapy, we can apply SPICA to interpret these data using our tumor-infiltrating T lymphocytes (TIL) reference atlas (Figure 2A). T cells from the two groups of patients, responders and non-responders before treatment, were projected separately, after subsampling to equalize the contribution of each sample. In the UMAP plots, we can see how TILs from each group of patients were projected onto the TIL reference atlas (Figure 2B and online at https://spica.unil.ch/projects/Bassez_2021). We can observe areas of high density of projected cells, such as in the CD8_EffectorMemory cell cluster in both groups. We can also observe some differences, such as the increased exhausted CD8 T cell population (CD8_Tex) in the responders group. Cell subtype composition can be examined more quantitatively in the ‘composition’ stacked barplots (Figure 2C). For instance, we observe higher frequency of CD8_Tex, follicular-helper (Tfh), and regulatory CD4 T cells (Treg) in the responder group, with lower frequencies of naive-like T cell states (CD8_NaiveLike and CD4_NaiveLike). Exact frequencies can be seen by hovering the mouse pointer over the corresponding barplot segment. These observation are in agreement with previous studies showing that responders to PD-1 blockade have higher relative amounts of CD8 Tex infiltrating their tumors, and a lower Treg to Tex ratio than non-responders (4–7). Radar plots are used to visualize and compare marker gene profiles of individual cell subtypes between projected samples and the reference (Figure 2D).

### Evaluating CD8 T cell diversity across tissues

The lymphocytic choriomeningitis virus (LCMV) infection is one of the best-studied model systems of viral infection, and has been instrumental in elucidating the biology of T cell responses. A recent study by Sandu et al. (8) used scRNA-seq to study CD8 T cell diversity in six different organs (spleen, blood, bone marrow, lymph node, liver and lung), to determine how the tissue microenvironment affects and shapes T cell phenotypes. In this case study, we applied SPICA to re-analyse the scRNA-seq data from Sandu et al. and study tissue-specific CD8 T cell heterogeneity in the context of a virus-specific CD8 T cell reference atlas.

**Figure 3.**
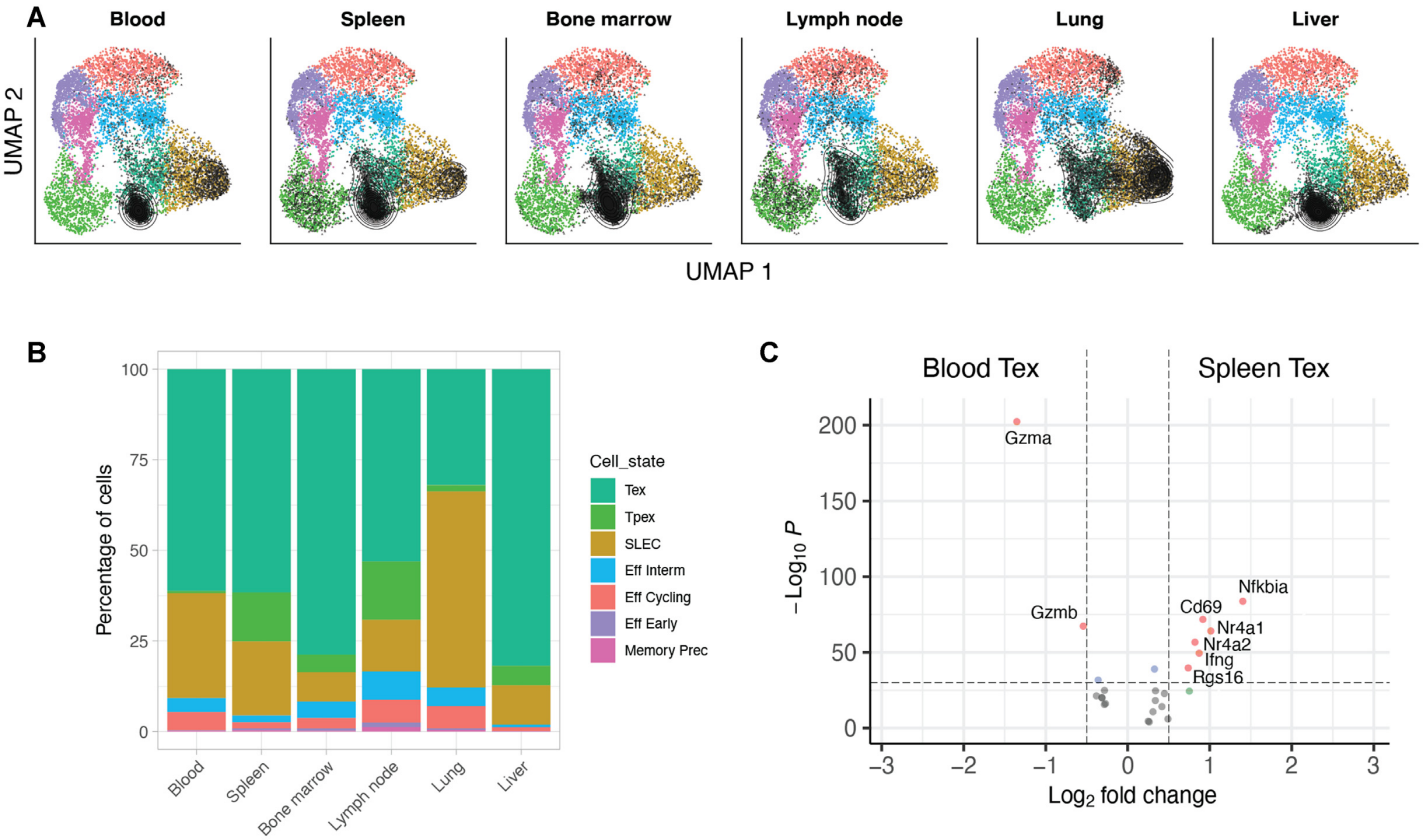
Case study of virus-specific CD8 T cells across tissues. (**A**) UMAP plots showing projection of query scRNA-seq data of CD8 T cells isolated from different tissues (by Sandu *et al.*) onto a reference atlas of virus-specific CD8 T cells. Black points represent projected query cells, contour lines represent the density of projected cells. (**B**) Cell subtypes composition (percentage) in each tissue/sample. (**C**) Volcano plot showing top differentially expressed genes between exhausted T cells (Tex) isolated from spleen compared to blood; only genes from the reference atlas with log_2_(fold-change) >0.25 are displayed. This case study can be explored online at https://spica.unil.ch/projects/Sandu_2020.

As expect in chronic infections, the majority of virus-specific T cells were predicted to be terminally exhausted (Figure 3A and online at https://spica.unil.ch/projects/Sandu_2020). However, there was also a level of heterogeneity between tissues in terms of subtype composition. For example, lung, spleen and blood showed increased percentages of short-lived effector cells (SLEC), whereas precursor exhausted (Tpex) T cells were enriched in lymph node and spleen compared to other tissues (Figure 3B). In addition to identifying these tissue-specific differences in T cell subtype composition, SPICA allows examining in higher resolution specific subtypes across samples and tissues. Differential expression analysis between specific T cell subtypes across tissues was executed within the SPICA portal. This analysis revealed that exhausted (Tex) T cells in spleen, where normally the viral load is higher, significantly overexpressed markers of antigenic stimulation (e.g. *Nfkbia*, *Nr4a1* and *Cd69*, Figure 3C) compared to Tex from blood, which instead upregulated granzymes (*Gzmb*, *Gzma*). By comparing transcriptional profiles of immune cells within the framework of a high-resolution reference atlas, SPICA allows streamlined exploration of tissue-specific adaptation signals.

## DISCUSSION

With the coming of age of single-cell technologies, an increasing number of valuable datasets are becoming available. Large repositories such as the Single Cell Portal at the Broad Institute (https://singlecell.broadinstitute.org/single_cell) or the Single Cell Expression Atlas at EBI (https://www.ebi.ac.uk/gxa/sc) are very useful resources to share and explore published single cell datasets. However, these portals do not curate datasets with consistent cell type definitions, and do not allow mapping new data to entries of the database. Comparison of studies and interpretation of new datasets is therefore challenging. In an effort to automate single-cell data analysis, the Azimuth app (https://azimuth.hubmapconsortium.org/) allows interpreting query data by reference-based mapping. However, the app only hosts whole-tissue low-resolution atlases. We have previously shown that cell-type specific reference atlases are essential to provide enough resolution to distinguish biologically important subtypes within each cell type, and that ProjecTILs (the algorithm behind SPICA) outperforms Azimuth for T cell subtype identification (3). This motivated us to create a specific public resource for immune cell state identification, hosting curated, cell type-specific reference atlases and a database of published studies analysed in the context of these atlases (Figure 1).

Though scRNA-seq data analysis requires bioinformatics expertise, reliable automated analysis pipelines are feasible when the computational methods have been designed for specific biological problems. By combining highly-specialized, curated reference cell atlases with robust algorithms for batch effect correction and reference atlas projection, the SPICA user interface enables seamless exploration of immune cell states across studies. SPICA will continue to incorporate high-quality reference atlases of immune cell types as these become available. The construction and annotation of accurate reference cell atlases is a challenging task, that requires expertise in a specific cell type. However, an atlas can potentially be applied to interpret an unlimited number of new studies, and the whole community can contribute to the analysis of published datasets. Thus, we expect the SPICA collection of case studies to grow quickly, contributing to understanding the diversity of immune cells in health and disease. In future versions of the portal, we envision being able to perform meta-analyses across projected datasets, and to enable similarity search between user data and the entries of the database. As the technology develops, it may be important to provide support for single-nucleus RNA-seq data, and to leverage information from T cell receptor and B cell receptor sequencing to explore the clonal structure of single-cell datasets.

## IMPLEMENTATION

SPICA is implemented as a Ruby on Rails (Model-View-Controller) application (version 5.2.4) running on a Docker container. The backend relational database is implemented with PostgreSQL. Project search on the main page is powered by an indexation with Sunspot, a port of Solr for Ruby on Rails. The frontend uses several javascript libraries, including jQuery.js, datatables.js and Plotly.js, to generate and export plots in generic formats. User authentication is handled by Devise rails library. Rails natively implements security features against Cross-Site Request Forgery and SQL injection. Analysis runs are dispatched with DelayedJob rails library on two queues (i) a queue for slow analyses (main projection pipeline) and (ii) a queue for fast analyses (e.g. radar plots generation, discriminant genes analysis). SPICA implements Websockets (ActionCable) to live monitor analysis progress and memory usage.

## DATA AVAILABILITY

The SPICA web portal is freely available at https://spica.unil.ch. The code for the algorithms used in SPICA is publicly available at https://github.com/carmonalab: STACAS for batch-effect correction and atlas construction (https://github.com/carmonalab/STACAS); UCell for filtering cells by signature scores (https://github.com/carmonalab/UCell); ProjecTILs for reference atlas projection (https://github.com/carmonalab/ProjecTILs).
